# Physicochemical aspects of inorganic nanoparticles stabilized in *N*-vinyl caprolactam based microgels for various applications

**DOI:** 10.1039/d0ra09327k

**Published:** 2021-01-04

**Authors:** Fatima Tahir, Robina Begum, Weitai Wu, Ahmad Irfan, Zahoor H. Farooqi

**Affiliations:** Institute of Chemistry, University of the Punjab New Campus Lahore 54590 Pakistan Zahoor.chem@pu.edu.pk zhfarooqi@gmail.com; State Key Laboratory for Physical Chemistry of Solid Surfaces, Collaborative Innovation Center of Chemistry for Energy Materials, The Key Laboratory for Chemical Biology of Fujian Province, Department of Chemistry, College of Chemistry and Chemical Engineering, Xiamen University Xiamen Fujian 361005 China; Research Center for Advanced Materials Science, Faculty of Science, King Khalid University Abha 61413 Saudi Arabia; Department of Chemistry, Faculty of Science, King Khalid University Abha 61413 Saudi Arabia

## Abstract

The vinyl caprolactam (VCL) based microgel system has become the center of great attention due to its versatile properties. Copolymerization of VCL with an ionic monomer imparts pH responsive properties into the microgel system in addition to thermo-sensitivity. Stimuli responsive behavior of VCL-based microgels makes them prospective and appealing candidates for practical applications covering the fields of drug delivery, catalysis and optical devices. In the last few years, VCL-based microgels have been used as microreactors and stabilizers for the synthesis and stabilization of inorganic nanoparticles to obtain hybrid microgels. The present review article provides a summary of the present-day progress of fabrication, stabilization, categorization and analysis of VCL-based microgels and their hybrids with different morphologies. The stimuli responsive properties and applications of VCL-based hybrid microgels have been reviewed critically. The remaining problems which need to be addressed have been pointed out for further advancement in this field.

## Abstract 2

The present review provides recent research progress of fabrication, stabilization, characterization and applications of *N*-vinyl caprolactam based microgels loaded with inorganic nanoparticles.

## Graphical abstract

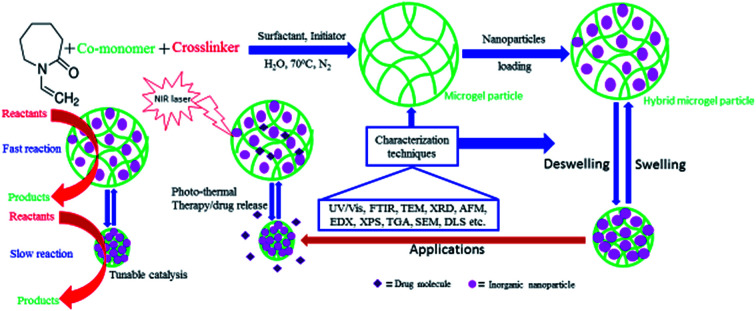

## 1.0 Introduction

Nanomaterials with controlled size, shape, and functionality has become a rapid growing research field owing to their distinctive optical, electrical, electronic and catalytic properties. Among various nanomaterials, inorganic nanoparticles have attracted much attention owing to their fascinating properties that differ from those of the corresponding bulk material. Their distinctive properties being dependent on size, shape and environment make them attractive for catalytic, sensing, antibacterial and biomedical applications.^1–4^ A characteristic property of aggregation associated with these nanoparticles reduces their surface to volume ratio and limits their applications. The problem of aggregation of nanoparticles is overcome by their deposition over various supports *i.e.* stabilizing agents.^5–7^ Polymer-based stabilizers are the most highlighted stabilizing systems to prevent aggregation of nanoparticles and have attracted attention as an effective template for preparing and stabilizing inorganic nanoparticles in solution.^8–11^ In this regard, environmentally responsive polymer microgels have been introduced as stabilizers for inorganic nanoparticles to obtain hybrid microgels with externally tunable properties.^12,13^ Microgels being cross-linked colloidal particles have capability either to swell or deswell in an appropriate solvent by slight variation in external stimuli. Among a wide variety of environmentally responsive microgels, thermo-responsive microgels have been widely reported.^14,15^ Thermo-responsive microgels show instant change in their size at a particular temperature called volume phase transition temperature (VPTT). Below VPTT, they are highly swollen and shrink rapidly at *T* ≥ VPTT.^16^ Copolymerization of an ionic monomer such as acrylic acid with thermosensitive monomer gives pH sensitivity to the temperature sensitive microgels.^17^ On the account of their stimuli responsive properties, microgels have attracted much attention as influential materials for a variety of applications in numerous fields including drug transfer, chemical and bio-sensing, micro-mechanical and optical devices, photonic crystals, environmental sciences, glucose sensing and catalysis.^18,19^ There are different types of polymeric microgels being reported with execution of smart behavior. Most investigated polymeric microgels functioning in aqueous medium are constructed using *N*-isopropylacrylamide (NIPAAm) and *N*-vinyl caprolactam (VCL) monomers. NIPAAm or VCL-based microgels have drawn special interest which has been dedicated to their thermo-sensitivity near to physiological temperature in aqueous environment.^20,21^ Among them, VCL based microgel system is more biocompatible than NIPAAm based microgel system following phase transition in the range of physiological temperature (32–38 °C) which is attained through modification in their structure by copolymerization with hydrophilic monomers.^22,23^ VCL is copolymerized with some ionic co-monomer *i.e.* weak acids like acrylic acid (AA),^24^ methacrylic acid (MA),^25^ itaconic acid (IA)^26^ or weak bases like vinyl pyridine (VP)^27^ to obtain microgels which undergo volume transitions against changes both in temperature and pH. VCL based microgel particles have been used as nano/micro templates for preparing hybrid nanomaterials^28,29^ Such hybrid nanomaterials offers a novel combination for the properties of both inorganic NPs and responsive polymeric materials for optical sensing, biomedical and catalytic applications.

Fabrication of VCL-based microgels and their hybrids for different applications is a subject of scientific literature of the last few years.^25,30–33^ Most of the publications on VCL based microgels deals with their biomedical applications. For example, Lou *et al.*^34^ employed dual responsive poly(*N*-vinylcaprolactam-*co*-undecenoic acid) [P(VCL-*co*-UA)] microgels as biocompatible materials for controlled release of drug *i.e.* doxorubicin. Wang *et al.*^35^ have reported the VCL based multi-responsive biodegradable microgels for biomedical applications *i.e.* drug delivery. Rao *et al.*^36^ studied controlled release of 5-Fluorouracil drug using poly(*N*-vinylcaprolactam) nanogels. Yerriswamy *et al.*^37^ also studied the release of same drug using poly(vinyl caprolactum-*co*-vinyl acetate) microspheres. Due to great attention towards biomedical applications of VCL based polymeric systems, only biomedical applications of this particular polymeric system has been reviewed in literature. Cortez-Lemus *et al.*^38^ have published a comprehensive review on a wide variety of architectures of *N*-vinylcaprolactam based systems which mainly deals with random copolymers, graft copolymers, block copolymers and physically/chemically crosslinked bulk polymeric systems without any detailed discussion on microgels and their hybrids. Mohammed *et al.*^17^ have reviewed the use of different *N*-vinyl caprolactam based polymers in novel drug delivery systems. Similarly, biomedical applications of *N*-vinyl caprolactam based polymers including their micelles and hydrogels have been reviewed by Liu *et al.*^39^ All aforementioned reviews are related to biomedical use of *N*-vinylcaprolactam based polymers not to the microgels. A mini review on a brief comparison of both synthesis and thermo-responsive behavior of poly(*N*-vinylcaprolactam) microgels/nanogels with poly(*N*-isopropylacrylamide) microgels/nanogels has been published by Ramos *et al.*^40^ As far as we know, synthesis, properties and applications of VCL based hybrid microgels have not been reviewed in literature.

Here in, synthesis, stabilization and characterization of VCL-based hybrid microgels for different applications have been reviewed critically. Section 1 highlights a brief overview of the presented topic. Section 2 and 3 cover methodologies used for synthesis of VCL based microgels and their hybrids respectively. Section 4 comprises of characterization techniques operated for analysis of both pure microgels and hybrid microgels. In Section 5, both temperature and pH responsive behavior of VCL based hybrid microgels have been discussed. Application of VCL based hybrid microgels in catalysis and biomedical field has been discussed in detail in Section 6 and 7. Section 8 high lightens summary and future aspects of VCL based microgels and hybrid microgels.

## 2.0 Synthesis of VCL based polymer microgels

The synthesis of VCL based microgels is fascinating not only for preliminary understanding of their properties but also for a variety of applications. Numerous strategies have been employed for preparing VCL based microgels including emulsion polymerization, precipitation polymerization and dispersion polymerization. Emulsion polymerization is a useful synthesizing technique which gives microgel particles with narrow particle size distribution. This technique involves the use of a surfactant to control the particle size and distribution of size. On one hand, it is a versatile method of microgel preparation while on the other hand; it faces the difficulty of complete removal of residual surfactant. This problem is overcome by surfactant-free polymerization which does not undergo any residual surfactant contamination. Precipitation polymerization is also used for preparation of VCL based microgels. In this case, both monomer and initiator are found to be soluble in reaction media and process of polymerization results in fabrication of insoluble polymer which leads the formation of stable dispersion of polymeric particles. Synthesizing VCL based microgels following above mentioned methods of preparation has been reported by various groups using different initiators and cross-linkers (Table 1). Laukkanen *et al.*^41^ made first attempt in the synthesis of VCL based microgels using both ionic and non-ionic initiators and surfactant by batch emulsion polymerization. Electrostatically, electrosterically and sterically stabilized polymer particles were prepared. Among these particles, the use of either ionic initiator or surfactant resulted in the formation of large aggregates at very moderate electrolyte conditions by electrostatically stabilized particles. During polymerization of VCL, co-monomers of different functionalities have been added which resulted in the particles with broad range of properties and applications. Thermo-sensitive microgels based on acetoacetoxyethyl methacrylate (AAEM) and VCL in water were synthesized using precipitation polymerization in surfactant-free environments by Boyko *et al.*^42^ Water-soluble 2,2′-azobis(2-methylpropioamidine) dihydrochloride (AMPA) and *N*,*N*′-methylenebis-acrylamide (MBA) were employed as cationic initiator and crosslinker respectively. Obtained microgel particles were found to have core–shell structure because of fast consumption of more reactive AAEM units during polymerization. Poly(vinylcaprolactam-*co*-acetoacetoxyethyl metacrylate) [P(VCL-AAEM)] microgel particles exhibited decreasing size upon increasing temperature of the medium. The same group of authors continued to report literature using the same P(VCL-AAEM) microgels as a micro-reactor for oxidative polymerization of pyrrole (Py),^43^ for depositing magnetite in the form of NPs,^33^ for incorporation of poly-pyrrole through selective microgel swelling in water–ethanol media^44^ and for fabrication of different nanoparticles.^45,46^ They also made an attempt of employing simple batch polymerization process for incorporating vinylimidazole (VIM) into the P(VCL-AAEM) in aqueous medium.^47^ This attempt resulted in synthesis of monodisperse and colloidally stable microgel particles having variable VIM contents. Resulted microgel particles have been found to exhibit both pH and temperature-sensitive behavior. Hantzschel *et al.*^48^ synthesized VCL based microgel particles using glycidyl methacrylate (GMA) as comonomer, AMPA as initiator and MBA as crosslinker under surfactant-free conditions using precipitation polymerization in water. The poly[vinylcaprolactam-*co*-(glycidyl methacrylate)] [P(VCL-GMA)] microgel particles were found to possess a spherical shape and heterogeneous core–shell structure with more compact GMA-rich core which is surrounded by a soft VCL-rich shell. Imaz *et al.*^49^ synthesized new microgel particles which were produced using VCL and poly(ethylene glycol) diacrylate (PEGDA) or MBA in a batch reactor. Resulting microgels were observed to possess the core–shell heterogeneous structure as reported in previous literature.^42,48^ Wang *et al.*^35^ synthesized VCL based microgels using VCL, methacrylic acid (MA) and polyethylene glycol methyl ether methacrylate (PEGMA) as monomers *via* precipitation polymerization. *N*,*N*-bis(acryloyl) cystamine (BAC) was employed as crosslinker and potassium persulfate (KPS) was employed as initiator while sodium bicarbonate (NaHCO_3_) was used in order to avoid hydrolysis of VCL during polymerization. Corporation of MA as pH-sensitive monomer and PEGMA being hydrophilic macromonomer were found to enhance the microgel stability by electrostatic and steric mechanisms. Till now, for synthesis of VCL based microgels, mostly cationic initiators were used which resulted in coagulation of particles. The use of anionic initiator like KPS for synthesizing VCL microgel led to VCL hydrolysis due to the acidic environment produced by KPS upon its decomposition in water. Such difficulties were overcome by Agrawal *et al.*^24^ They prepared (*N*-vinylcaprolactam-acetoacetoxyethyl methacrylate-acrylic acid) P(VCL-AAEM-AA)] microgels using precipitation polymerization technique. This group followed the same synthesis procedure as reported by Pich *et al.*^43^ For synthesis, anionic initiator 2,2′-azobis [*N*-(2-carboxyethyl)-2-methylpropionamidine] (ACMA) was used rather than cationic initiator 2,2′-azobis(2-methylpropionamidine) dihydrochloride (AMPA) as reported in previous studies.^34,42–44,47^ The use of the anionic initiator resulted in prevention of coagulation during the particle nucleation. The use of anionic initiator *i.e.* ACMA resulted into stable colloidal dispersions with better yield.

**Table 1:** *N*-Vinylcaprolactam based microgels synthesized by different methods along with monomers, crosslinker and initiator used

Entry	Microgel system	Method	Monomers	Cross-linker	Emulsifier	Initiator	References
1	P(VCL)	Batch emulsion polymerization	VCL	MBA	SDS, PEO-R-MA	KPS, VA-086	41
	P(VCL-AAEM)	Precipitation polymerization	VCL, AAEM	MBA	—	AMPA	34,42 and 43
3	P(VCL-AAEM-VIM)	Batch polymerization	VCL, AAEM, VIM	MBA	—	AMPA	47
4	P(VCL-GMA)	Precipitation polymerization	VCL. GMA	MBA	—	AMPA	48
5	P(VCL-*co*-PEGDA)	Emulsion polymerization	VCL, PEGDA	MBA, PEGDA	SDS	KPS	49
6	P(VCL-ss-MA)-PEG	Precipitation polymerization	VCL, MA, PEG	BAC	SDS	KPS	35
7	P(VCL-AAEM-AA)	Precipitation polymerization	VCL, AAEM, AA	MBA	—	ACMA	24
8	P(VCL-*co*-MAA)	Precipitation polymerization	VCL, MA	MBA	SDS	KPS	17
8	P(VCL-α-CD)	Precipitation polymerization	VCL, AAEM	MBA	CTAB	AMPA	46
9	P(VCL-*co*-4VP)	Free radical emulsion polymerization	VP	MBA	SDS	KPS	27

## 3.0 Synthesis of VCL based microgel stabilized nanoparticles

Various nanoparticles have been incorporated inside the microgel systems resulting in hybrid microgels with properties of both organic and inorganic components. Various strategies have been applied for formation of hybrid microgel systems which involves either incorporation of nanoparticles by developing interactions between nanoparticles and microgels or by fabrication of nanoparticles inside the microgel system. *In situ* synthesis of inorganic nanoparticles is widely used approach to obtain hybrid microgel system. Synthesis of various hybrid microgel systems for various applications is a subject of intense research and has been reviewed by us^50,51^ and others^52,53^ but here in, our discussion is restricted to synthesis of VCL based hybrid microgels. The nanoparticles incorporated inside the VCL based microgel systems can be classified into various categories which have been discussed below.

### 3.1 Metal nanoparticles

**Fig. 1:**
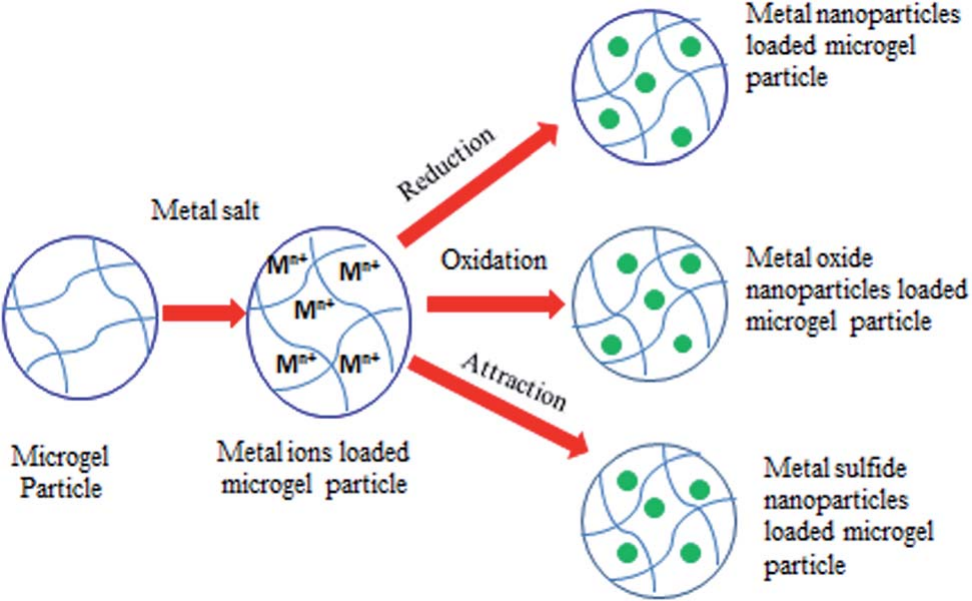
Schematic illustration of fabrication of metal nanoparticles, metal oxide nanoparticles and metal sulfide nanoparticles within VCL based microgels.

Fabrication of metal nanoparticles loaded polymer microgels has been extensively reported in literature^54,55^ but our discussion is restricted to metal nanoparticles stabilized in VCL based microgels. Metal nanoparticles are generally synthesized by reduction of metal salts within the sieves of VCL based microgels (Fig. 1).

**Fig. 2:**
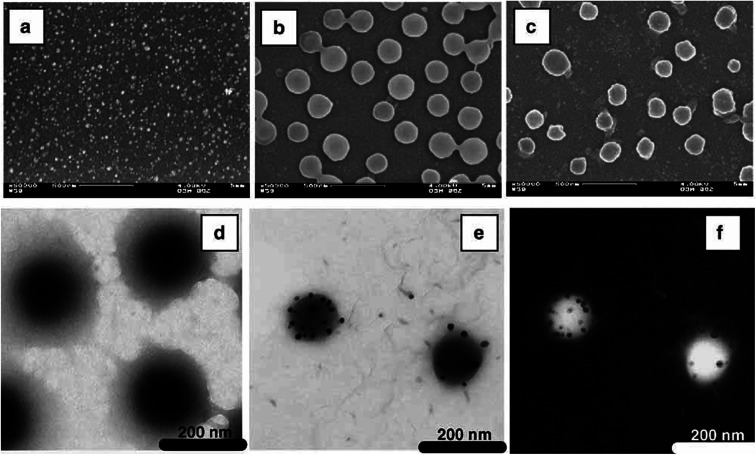
SEM images of silver nanoparticles (a), P(VCL-AAEM) microgel particles (b) and Ag-P(VCL-AAEM) hybrid particles (c) and TEM images of P(VCL-AAEM) microgel particles (d) and Ag-P(VCL-AAEM) hybrid particles (e) along with electronic spectroscopic image (f). These images have been reproduced with permission from ref. 45. Copyright©2006 Wiley and Sons.

Fabrication of gold and silver nanoparticles in VCL based microgels have been widely reported by *in situ* reduction strategy.^24,45,46^ Pich *et al.*^45^ have fabricated silver nanoparticles (AgNPs) in P(VCL-AAEM) microgel particles using *in situ* reduction methodology. P(VCL-AAEM) microgel particles were first synthesized and then chemical reduction of silver nitrate (AgNO_3_) by sodium borohydride (NaBH_4_) was followed for *in situ* incorporation of silver nanoparticles (Ag NPs) inside the microgel system. Content of silver nanoparticles were increased with rise of original concentration of AgNO_3_ but beyond maximum incorporation limit, AgNPs remain outside the microgel particles. Heterogeneous structure of microgel particles (high crosslinking density in core region in comparison to shell region) led to prevent deep penetration of AgNPs. Effect of loading various contents of silver nanoparticles on structure and properties of the microgel system was investigated which will be discussed in Section 4. Silver-poly[vinylcaprolactam-*co*-(acetoacetoxyethyl methacrylate)] [Ag-P(VCL-AAEM)] hybrid microgel particles were successfully immobilized on glass surface to obtain an easily recoverable and re-usable catalytic system. Same group has reported synthesis of gold nanoparticles (AuNPs) in the same microgel system [P(VCL-AAEM)] using the same methodology as described above for the fabrication of silver nanoparticles in P(VCL-AAEM).^56^ The chemical reduction of chloroauric acid (HAuCl_4_) salt loaded into P(VCL-AAEM) microgel was exercised by using trisodium citrate as reductant in aqueous medium to get gold-poly[vinylcaprolactam-*co*-(acetoacetoxyethyl methacrylate)] [Au-P(VCL-AAEM)] hybrid microgel system for catalytic applications. Au nanoparticles were not homogeneously distributed in P(VCL-AAEM) particles due to heterogeneous structures of microgel particles. Most of the nanoparticles were found in outer shell region (VCL-rich loosely crosslinked region) instead of tightly crosslinked core region (AAEM-rich region) of the microgels. Such kind of hybrid systems has a great significance in case of their use in catalysis which will be discussed in later sections. In both above mentioned hybrid microgel systems, metal nanoparticles were not homogeneously distributed within the microgel particles as shown in Fig. 2. The Fig. 2(a–c) gives SEM images of Ag nanoparticles (a), P(VCL-AAEM) microgel particles (b) and Ag-P(VCL-AAEM) hybrid microgel particles while Fig. 2 (d–f) shows TEM images of P(VCL-AAEM) microgel particles (d) and Ag-P(VCL-AAEM) hybrid particles (e) along with electron spectroscopic image of hybrid microgel particles (f).

**Fig. 3:**
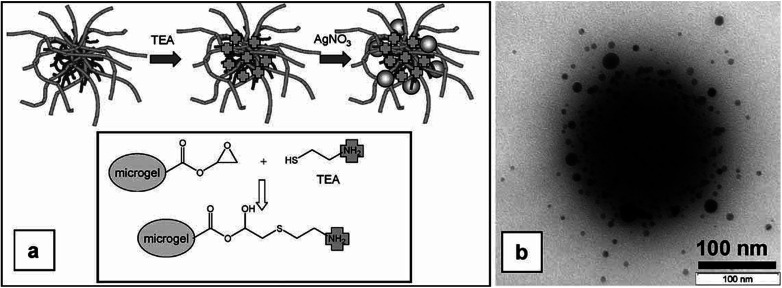
Modification of poly(*N*-vinylcaprolactam-*co*-glycidyl methacrylate) P(VCL-GMA) microgel system with 2-aminoethanthiol (TEA) to obtain modified P(VCL-GMA) microgel particles (a) and TEM image of Ag-P(VCL-GMA) hybrid microgels (b). These images have been reproduced with permission from ref. 48. Copyright©2009 Wiley and Sons.

Homogeneous distribution of metal nanoparticles in VCL based polymer microgels was successfully achieved by Hantzschel *et al.*^48^ who synthesized poly(*N*-vinylcaprolactam-*co*-glycidyl methacrylate) P(VCL-GMA) microgel system and modified it with 2-aminoethanthiol (TEA) to obtain modified P(VCL-GMA) microgel particles. The modification led to incorporation of amino groups in interior of microgels by reaction of epoxy functionalities of GMA with thiol functionalities of TEA. The amino groups acted as chelating sites to keep AgNPs in the interior of microgels. The modified microgel system was used as micro-container for the fabrication of microgels using *in situ* reduction strategy as described above. Ag NPs were successfully incorporated into interior of the modified microgel particles without any aggregation over the time. No significant number of Ag NPs was detected outside the microgel network. Fig. 3 gives schematic illustration of fabrication of silver nanoparticles in modified microgels (a) and TEM image of hybrid microgels (b).

Hou *et al.*^27^ incorporated AuNPs into poly(*N*-vinylcaprolactam-*co*-4-vinyl pyridine) P(VCL-*co*-4VP) microgels by chemical reduction of HAuCl_4_ loaded into P(VCL-*co*-4VP) polymer network by NaBH_4_ in aqueous medium. 4-VP having pyridine rings helps to stabilize the AuNPs inside the polymer network. AuNPs obtained inside the network were spherical and well-distributed throughout the network. The homogenous distribution of AuNPs within the VCL based microgels without using any external (usually toxic) reducing agent have been reported by Jia *et al.*^46^ who carried out copolymerization of VCL and AAEM by precipitation polymerization in the presence of α-cyclodextrin (α-CD) and then used α-CD modified P(VCL-AAEM) microgels as nano-reactors for the incorporation of AuNPs. Addition of HAuCl_4_ solution into α-CD modified P(VCL-AAEM) microgel dispersion in the presence of sodium hydroxide (NaOH) led to color change which was attributed to formation of Au nanoparticles by reduction of metal precursor by functionalities of VCL and α-CD. The Au-α-CD-P(VCL-AAEM) hybrid microgel system with uniform nanoparticles distribution in interior of polymeric network prepared by facile and green approach was found as an excellent catalytic system with thermally tunable catalytic activity which will be discussed in Section 4 in detail. The confinement of Au nanoparticles in central region of VCL based microgels may give them more stability. Agrawal *et al.*^24^ reported fabrication of gold nanoparticles (AuNPs) in central region of P(VCL-AAEM-AA) microgel particles by *in situ* reduction of HAuCl_4_ without using any external reducing agent. AuNPs were strictly localized in core region of the microgels due to presence of high content of AAEM units in central region in comparison to shell region of the microgels. It was expected that β-diketonic functionalities of AAEM are responsible for reduction of gold salt by electron rich oxygen atoms because of keto–enol tautomerization but more investigations are needed to fully realize the mechanism of reduction of Au salt by P(VCL-AAEM-AA) microgels and confinement of Au nanoparticles in the central region. Some additional inorganic material already present in VCL based microgels may enhance metal nanoparticles anchoring ability of the microgels. Contin *et al.*^57^ have fabricated composite microgel particles of clay and P(VCL-AAEM) polymeric network and then loaded cationic precursors of palladium (Pd), platinum (Pt) and Au to prepare Pd-clay-P(VCL-AAEM), Pt-clay-P(VCL-AAEM) and Au-clay-P(VCL-AAEM) hybrid microgels. It is believed that clay material has ability to exchange their cations with cationic precursors of metal which may be reduced to metal nanoparticles using suitable reducing agent. Moreover *in situ* generated metal nanoparticles are adsorbed on the surface of clay particles. Adsorption of metal nanoparticles on clay particles already loaded in P(VCL-AAEM) system gives extraordinary stability to metal nanoparticles. The chances of leaching of metal nanoparticles during catalysis by such kind of hybrid microgels are negligible due to strong interaction between clay and metal particles. Siirila *et al.*^58^ decorated P(VCL) nanogels surfaces with gold (Au) nanoparticles by mixing synthesized Au nanoparticles with P(VCL) based nanogels being synthesized by precipitation polymerization. Synthesized Au nanoparticles were protected with a mixture of ligands of 11-azidoundecanethiol (AZT) and 11-mercaptoundecanoicacid (MUA) and bounded to propargyl functionalized P(VCL) based nanogels. Acidic groups on the surfaces of both Au nanoparticles and P(VCL) based nanogels resulted in stabilization of particle dispersions above VPTT of P(VCL). Fabricated Au nanoparticles were observed to be localized in soft surface layer of the nanogels and found to possess truncated octahedron shape.

### 3.2 Bimetallic nanoparticles

Bimetallic nanoparticles may be loaded into polymer microgels using *in situ* reduction methodology as described for fabrication of monometallic nanoparticles in Section 3.2 and has been reported in literature for *N*-isopropylacrylamide based hybrid microgels.^59–61^ To the best of our information, fabrication of bimetallic nanoparticles in VCL based microgels using *in situ* reduction methodology has not been reported in literature. However separately prepared bimetallic nanoparticles have been loaded into VCL based microgels to obtain a hybrid material with fascinating properties. For example, Wiemer *et al.*^32^ have obtained hybrid system consisting of poly(*N*-vinylcaprolactam) microgels loaded with superparamagnetic bimetallic FePt nanoparticles using solvent exchange method. FePt nanoparticles were synthesized in dioctylether by using iron (acetylacetonate) and platinum (acetylacetonate) as precursors under argon purge and were found to be soluble in tetrahydrofurane (THF). After synthesis, FePt nanoparticles dispersion in THF was charged with poly(*N*-vinylcaprolactam) particles. Exchanging solvent from THF to water, forced hydrophobic FePt bimetallic nanoparticles into polymeric network resulting in FePt nanoparticles VCL based hybrid microgels.

### 3.3 Metal oxide nanoparticles

**Fig. 4:**
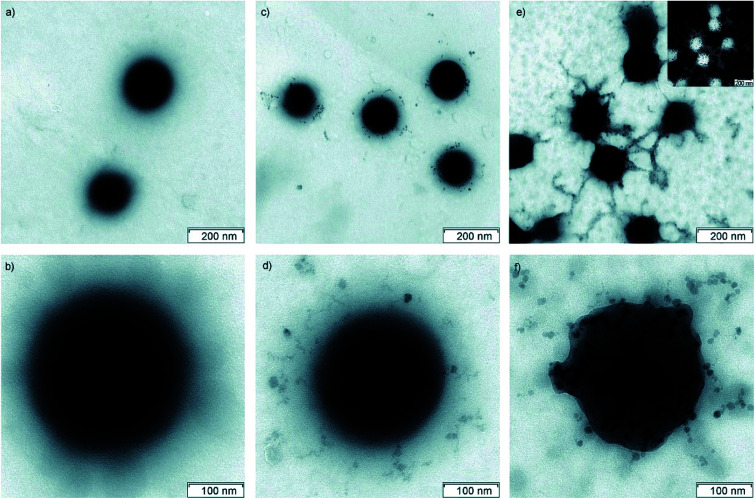
TEM images of poly(vinylcaprolactam-*co*-acetoacetoxyethyl methacrylate-*co*-vinylimidazole) [P(VCL-AAEM-VIM)] microgel particles with no Fe_3_O_4_ contents(a and b), Fe_3_O_4_-P(VCL-AAEM-VIM) hybrid microgel particles with Fe_3_O_4_-8.41 weight% (c and d), Fe_3_O_4_-P(VCL-AAEM-VIM) hybrid microgel particles with Fe_3_O_4_-15.35 weight% (e and f) and EDX mapping image of iron (inset of e). These images have been reprinted with permission from ref. 62. Copyright©2007 Wiley and Sons.

Loading of metal oxide nanoparticles into VCL based microgels results in fabrication of hybrid microgels with fascinating properties of metal oxide as well as microgel system and leads to a wide range of applications (Fig. 1). For example loading of magnetite (Fe_3_O_4_) nanoparticles into VCL based microgels gives hybrid microgels with magnetic properties, temperature sensitivity and colloidal stability. Pich *et al.*^33^ prepared hybrid microgels by direct deposition of magnetite nanoparticles (Fe_3_O_4_ NPs) inside the P(VCL-AAEM) microgels. Different amounts of iron(ii) chloride (FeCl_2_) and iron(iii) chloride (FeCl_3_) with constant ratio (1 : 2) were added into the P(VCL-AAEM) dispersion under N_2_ atmosphere followed by addition of aqueous solution of ammonium hydroxide (NH_4_OH) to fabricate Fe_3_O_4_ NPs inside the microgels. Resulting hybrid microgels were found to be stable over a wide temperature range and showed thermo-sensitivity and magnetic properties. Bhattacharya *et al.*^62^ prepared hybrid microgels by incorporating magnetite nanoparticles inside poly(vinylcaprolactam-*co*-acetoacetoxyethyl methacrylate-*co*-vinylimidazole) [P(VCL-AAEM-VIM)] microgels. Fabrication of magnetite nanoparticles was followed by reaction of different amounts of FeCl_3_ and FeCl_2_ in constant ratio with aqueous solution of NH_4_OH under nitrogen purge in the presence of microgel particles containing VIM (4.91 mol%). The spherical microgel particles (Fig. 4(a and b)) were successfully loaded with Fe_3_O_4_ nanoparticles (Fig. 4(c and d)). The increase in content of magnetite nanoparticles in Fe_3_O_4_-P(VCL-AAEM-VIM) hybrid microgels results in entanglement of magnetite components and deformation of shape of microgel particles (Fig. 4(e and f)).

Resultant hybrid microgels were found to possess considerable temperature and pH sensitivity with high magnetic response. Corresponding new hybrid microgels found to exhibit colloidal stability in water and superparamagnetic characteristics. Agrawal *et al.*^63^ reported fabrication of ZnO nanoparticles in P(VCL-AAEM) microgels for photo-catalysis and UV shielding applications. Required amount of zinc acetate dihydrate [Zn(CH_3_COO)_2_·2H_2_O] was dissolved in 2-propanol followed by addition of P(VCL-AAEM) dispersion. Then NaOH solution was poured into the reaction mixture to obtain ZnO-P(VCL-AAEM) hybrid microgels. Hydrolysis of Zn(CH_3_COO)_2_·2H_2_O results in positively charged zinc complex which is attracted into the microgel network for fabrication of ZnO nanoparticles. The β-diketone functionalities of AAEM and amide group of VCL form co-ordinate bond with Zn^2+^ to stabilize ZnO nanoparticles.

### 3.4 Metal sulfide nanoparticles

*N*-Vinylcaprolactam based colloidal particles have also been employed for fabrication and stabilization of metal sulfide nanoparticles (Fig. 1). Peng *et al.*^25^ fabricated hybrid nanostructures by *in situ* thermal synthesis of copper sulfide (CuS) nanocrystals within poly(*N*-vinyl caprolactam-*co*-methacrylic acid) [P(VCL-*co*-MAA)] microgels. CuCl_2_ aqueous solution was added into the P(VCL-*co*-MAA) microgel dispersion under pH > p*K*_a_ of MAA to load Cu^2+^ ions into polymer network through electrostatic attraction between COO– groups and metal ions. Ethanol was added into the microgel dispersion before addition of Na_2_S solution. S^2−^ ions diffuse from bulk region to polymer network and are attached with Cu^2+^ ions already present inside the microgels. Temperature was raised to 70 °C to initiate CuS crystallization which led to the formation of plake-like covellite CuS nanocrystals. Resulting fabricated hybrid nanostructures have been found to possess better temperature sensitivity, adjustable loading capacity and excellent colloidal stability. Pich *et al.*^64^ prepared hybrid microgels by the direct deposition of zinc sulfide (ZnS) nanoparticles inside the P(VCL-AAEM) microgels by ultra-sonication method. Different amounts of zinc acetate (ZnAc) and thioacetamide (TAA) in molar ratio of 1 : 1 were added under ultrasonic agitation at 5 °C to fabricate ZnS nanoparticles inside microgels. Respective ZnS nanoparticles get stabilized due to hydrophobicity possessed by interaction of nanoparticles with polymeric network. Resulting hybrid microgels exhibited temperature-responsive properties, high ZnS contents and good colloidal stability.

### 3.5 Miscellaneous nanoparticles

Hantzschel *et al.*^65^ have reported fabrication of europium-doped lanthanum fluoride (Eu-LaF_3_) nanoparticles in P(VCL-GMA) microgels. For this purpose, 2-aminoethyl dihydrogenphosphate (AEP) was dispersed in aqueous solution of ammonium hydroxide. The aqueous solution of required amounts of Eu(NO_3_)_3_·6H_2_O and La(NO_3_)_3_·6H_2_O was added into the reaction mixture followed by addition of NaF to obtain Eu-LaF_3_ nanoparticles stabilized by AEP. Required amount of Eu-LaF_3_ nanoparticles was added into already prepared P(VCL-GMA) microgel dispersion to fabricate hybrid microgel system. The amino functionalities of VCL and epoxy groups of GMA present in microgels were responsible for stability of nanoparticles within microgels.

## 4.0 Characterization

VCL based hybrid microgels have been characterized by UV-visible spectroscopy (UV-Vis), Fourier transform infrared microscopy (FTIR), Transmission electron microscopy (TEM), X-ray diffraction technique (XRD), Atomic force microscopy (AFM), Atom absorption spectroscopy (AAS), Laser light scattering measurements, Energy-dispersive X-ray (EDX), X-ray photoelectron spectroscopy (XPS), Thermogravimetric analysis (TGA), Scanning electron microscopy (SEM) and Dynamic light scattering (DLS). The summary of techniques used for characterization of various VCL based microgels loaded with different inorganic nanoparticles reported in literature has been presented in Table 2. Theoretical background of characterization techniques is beyond the scope of present review. Only use of techniques in analysis of VCL based hybrid microgels has been described in this section of the review.

**Table 2:** Characterization techniques used for VCL based microgels and hybrid microgels

Pure microgel system	Hybrid microgel system	Techniques	Description of the system on the basis of techniques used	Ref.
P(VCL)		DLS, CE	Electrostatically, sterically and electrosterically stabilized P(VCL) microgels were temperature sensitive but sterically stabilized one was found to be more stable against electrolyte.	39
P(VCL-AAEM)		FTIR, TGA, LLS, SEM	Particle size decreased with increased particle heterogeneity. Microgel particles were found to be stable.	42
	P(VCL-AAEM-PPy)	SEM, DLS, FTIR	Strong interaction of PPy particles with VCL/AAEM network stabilized PPy inside microgel network and led to decreased hydrodynamic radius.	43
	Fe_2_O_3_-P(VCL-AAEM)	DLS, SEM, AFM, TGA,	VCL/AAEM microgels were stable in swollen state in water and polydisperse in dried state.	33
			Decrease in hydrodynamic radius followed by increased radius for composite microgels was observed with less spherical partical core. Magnetite particles were found to be stabilized inside microgel with small amount in aqueous phase.	
	Ag(VCL-GMA)	DLS, SEM, TEM, AAS, cryo-TEM	Ag nanoparticles were found to be stabilized inside microgel network and randomly distributed in outer layer without aggregation.	48
	Au-(VCL-AAEM-AA)	DLS, TEM, TGA, XPS	Decrease in hydrodynamic radius upon loading of gold nanoparticles and increased amount of nanoparticles inside microgel was observed indicating successful incorporation in core region.	42
	Ag-(VCL-AAEM)	DLS, SEM, EDX, TGA, UV-Vis spectroscopy	Hydrodynamic radius decreased upon fabrication of Ag nanoparticles resulting into roughed microgel surface with homogenous distribution of nanoparticles. Composites were found more thermal stabile. Surface plasmon resonance band was appeared at 430 nm for Ag nanoparticles.	45
	Au-(VCL-AAEM)	DLS, TEM, SEM	Hydrodynamic radius was decreased and hybrid microgels were found to be stable at higher contents of Au nanoparticles which were found to be localized in outer region.	56
	Au-P(VCL-*co*-4VP)	DLS, TEM, TGA, FTIR	Both microgel particles and Au nanoparticles found to be spherical in shape. Both pure and hybrid systems were thermally stable under 400 °C. Decreased hydrodynamic radius was observed after fabrication of nanoparticles.	62
	ZnO-P(VCL-AAEM)	DLS, TEM, EMI, TGA, UV-Vis spectroscopy	ZnO nanoparticles remained spherical and homogenous in size after fabrication and were aggregated inside microgel in dried state. Decreased hydrodynamic radius followed by an increase was observed. After fabrication, composite particles showed absorption in range 357–362 nm.	63

DLS is one of the widely used techniques for analysis of VCL based hybrid microgels. The value of hydrodynamic radius (*R*_h_) of the microgels is measured by DLS measurement. The pH and temperature responsive behavior of VCL based hybrid microgels has been investigated by measuring *R*_h_ value as a function of pH and temperature of the medium using DLS. Size distribution of VCL based microgel particles is highly important for their colloidal stability and their applications in various fields. DLS is also used for determination of size distribution of the microgels. The variation in *R*_h_ value of the microgel upon loading of inorganic nanoparticles is also investigated by DLS. UV-Vis spectroscopy is used to check the presence of plasmonic nanoparticles (Ag, Au and CuS *etc.*) loaded to VCL based microgels. Some information regarding size, size distribution and shape of plasmonic nanoparticles can be also obtained from UV-visible spectra of hybrid microgels.^54^^.^ AFM is used to study the variation in surface properties of nanoparticles loaded microgels being placed on some substrate under different environmental conditions. FTIR is used for identification of functional groups and bonding between inorganic nanoparticles and functionalities of VCL based microgels. XRD is used to investigate either amorphous or crystalline behavior of VCL based hybrid microgels. It is also used for determination of crystallite size of inorganic nanoparticles loaded into VCL based polymeric system. EDX is used to identify nature and composition of inorganic materials loaded into VCL based microgels. Determination of size, shape, distribution and morphology of inorganic nanoparticles are carried by TEM while SEM is used to study the surface morphology of nanoparticles and VCL microparticles. AAS is used to determine the metal content in VCL based hybrid microgels. TGA is used for examination of thermal stability of VCL based microgels and hybrid microgels. It is also used for determination of inorganic content in VCL based microgels.

## 5.0 Properties of VCL based hybrid microgel systems

VCL based hybrid microgels are fascinating colloidal materials having properties of both inorganic and organic components in a single system. This unique feature makes them potential candidates for various biomedical, environmental and catalytic applications. Properties of VCL based hybrid system may be classified into (a) properties due to microgel component and (b) properties due to inorganic nanoparticles components.

### 5.1 Properties of VCL based hybrid microgels due to organic component

#### 5.1.1 Temperature responsive properties of VCL based hybrid microgels

Thermo-responsive microgel particles exhibit sudden change in their size on changing the temperature of medium. Such behavior of microgels is termed as their thermo-sensitivity. P(VCL) microgels are commonly known thermo-responsive microgels. P(VCL) colloidal particles exist in swollen state at low temperature and shrunken state at high temperature. They show sudden change in their hydrodynamic radii at a particular temperature which is known as volume phase transition temperature (VPTT). The value of VPTT of P(VCL) microgels in water is 32 °C.^22,23^ The value of VPTT of VCL based microgels depends upon various factors including solvent, co-monomer used, pH of the medium, ionic strength of the medium and any additional moieties present in the polymer network. The volume phase transition (VPT) in the microgel occurs due to breakage of hydrogen bonding between water molecules and various functionalities of VCL based microgels. The temperature, at which VPT occurs, depends on the hydrophobic/hydrophilic balance of the microgel particles. Thermo-responsive behavior of VCL based microgels under various conditions has been widely investigated in last decade. An overview of the studies reported has been presented in this section. Boyko *et al.*^42^ investigated the effect of feed content and temperature of the medium on the value of hydrodynamic radius of P(VCL-AAEM) microgel particles. Value of hydrodynamic radius (*R*_h_) of copolymer microgel particles was found to be decreased with increase of AAEM mol% in microgels at 20 °C. A slight shift in value of VPTT (from 32 °C to 28 °C) of the copolymer microgels was observed between transition temperatures of microgel by varying the content of AAEM from 1.25 to 5 mol% which may be caused by increase of content of hydrophobic monomer in microgel system. It is well known that the value of VPTT of VCL based microgels can be tuned by changing the co-monomer and its feed content in microgels. VPTT temperature may be shifted to higher temperature by copolymerization of VCL with hydrophilic monomer and its feed content. An excellent piece of work in this regard, has been presented by Pich *et al.*^47^ who fabricated P(VCL-AAEM) microgels and reported that this system undergoes volume phase transition at 28 °C and then prepared VCL based polymer microgels by polymerization of VCL, AAEM and vinylimidazole (VIM) under similar conditions. The addition of VIM as one of the co-monomers does not only increase the hydrodynamic radius of the microgels but also shift the value of VPTT to higher temperature due to hydrophilic character of VIM. The values of *R*_h_ and VPTT were found to increase with increase of mol% of VIM in P(VCL-AAEM-VIM) microgels. VIM does not only shift the value of VPTT to higher temperature but also gives pH sensitivity to the microgel system. Therefore value of *R*_h_ and VPTT were found to be dependent of pH of the medium and were found to be higher at low pH value as compared to those at high pH values of the medium. The value of VPTT of P(VCL-AAEM-VIM) microgels was found to be 55 °C at pH = 4 due to increase in hydrophilicity (strong interaction between water molecules and polymer functionalities) of P(VCL-AAEM-VIM) microgels caused by protonation of VIM groups at this pH value. Effect of addition of co-monomers, their nature and contents has been widely reported and can be seen in literature^66–69^ but our interest is limited to thermo-sensitivity of inorganic nanoparticles loaded VCL based microgels. A lot of work on thermo-responsive behavior of VCL based hybrid microgels has been reported by Pich and co-workers.^34,43,44^ For example, Pich *et al.*^45^ studied thermo-sensitivity of Ag–P(VCL-AAEM) hybrid microgels and P(VCL-AAEM) microgels and observed that microgel system does not lose thermo-sensitivity after loading of silver nanoparticles into microgels (from 1.0% to 15% Ag NPs) but upon further increase in AgNPs contents results in loss of thermo-sensitivity of the microgels. The values of *R*_h_ and VPTT temperature of the Ag–P(VCL-AAEM) were found to be decreased with increase in Ag content in microgels due to strong chelating interaction between Ag nanoparticles and functionalities of the microgels. Pich *et al.*^56^ have also reported similar thermosensitive behavior of P(VCL-AAEM) microgel upon loading of AuNPs as was observed in case of loading of AgNPs in the same system. Jia *et al.*^46^ studied the thermo-responsive behavior of α-cyclodextrin functionalized P(VCL) microgels loaded with gold nanoparticles (PVCL-α-CD-Au). The value of VPTT of Au loaded microgels was found to be same as that of P(VCL-α-CD) (27.5 °C). The swelling-deswelling behavior of P(VCL-α-CD) microgels was not affected by loading of Au nanoparticles into P(VCL-α-CD) network. However volume phase transition of Au–P(VCL-α-CD) was found to more sharper than that in case of pure microgels which was probably due to adsorption of α-CD on the surface of Au nanoparticles because some α-CD molecules become unavailable for crosslinking in the presence of Au nanoparticles. Pich *et al.*^33^ studied the effect of magnetite NPs on the value of *R*_h_ and thermo-responsive behavior of microgels using DLS measurements. The value of *R*_h_ of microgels were found to decrease with increase of content of Fe_3_O_4_ (from 1.0 to 7%) inside the microgels which may be attributed to shrinkage of polymeric network caused by complexation between Fe_3_O_4_ and functionalities of microgels. Interestingly the value of *R*_h_ was observed to increase with further increase of Fe_3_O_4_ from 7.0 to 16%) which was attributed to repulsive forces between charged magnetite nanoparticles loaded into microgel system. In spite of loading of metal nanoparticles into P(VCL-AAEM) microgels, magnetite NPs loading did not affect thermo-responsive behavior of Fe_3_O_4_–P(VCL-AAEM) microgels significantly. However slight shift in VPTT was observed upon loading of Fe_3_O_4_ nanoparticles into microgels due to interaction of iron oxide particles with polymer chains of microgels. Exactly similar trend of metal oxide content dependence of *R*_h_ and VPTT values of P(VCL-AAEM) microgels has been reported by Agrawal *et al.*^63^ who studied the temperature sensitive properties of ZnO–P(VCL-AAEM) hybrid microgels in aqueous medium using DLS measurements. Siirila *et al.*^58^ reported temperature sensitive property of P(VCL)-Au nanoparticles using DLS measurement. P(VCL)-Au nanoparticles exhibited thermosensitive behavior and hydrodynamic radius was found to increase at all temperatures on comparing pure P(VCL) nanogels with P(VCL)-Au nanoparticles. Difference in hydrodynamic size between pure P(VCL) nanogels and P(VCL)-Au nanoparticles was attributed to hydrophilic character of AuNPs at measuring pH of 7.4. Peng *et al.*^25^ studied thermo-responsive properties of P(VCL-MAA)-CuS hybrid microgels having feeding Cu^2+^/–COOH molar ratios of 20 : 2 as a function of pH of the medium. Increase in pH values have been found in shifting of cloud points of hybrid microgels to higher temperatures which was attributed to increased ionization of carboxyl groups at higher pH values.

#### 5.1.2 The pH responsive behavior of VCL based microgels

The variation in hydrodynamic radius of microgels with change in pH of the medium is called their pH sensitivity or pH responsive behavior. VCL moiety has been found as non-responsive to pH change over the wide range of pH of the medium. However copolymerization of VCL with some ionizable monomer in the presence of suitable cross linker results in fabrication of pH responsive polymer microgels. The copolymerization of VCL with acrylic acid,^24^ vinyl imidazole,^47^ 4-vinyl pyridine^27^ to obtain pH responsive microgels has been documented in literature. Pitch *et al.*^47^ studied the pH responsive behavior of P(VCL-AAEM-VIM) microgels in aqueous medium. Maximum swelling in microgel particles (maximum *R*_h_ value) was observed at pH = 4 due to electrostatic repulsion of positively charged VIM groups that exist completely in protonated form at this particular pH value. The decrease in *R*_h_ value was observed at pH < 4 due to increase of ionic strength caused by addition of HCl (a reagent used for adjustment of pH). Effect of pH on value of hydrodynamic radius of both P(VCL-AAEM) and P(VCL-AAEM-VIM) microgels was compared with each other. In contrast to P(VCL-AAEM-VIM) microgels, P(VCL-AAEM) microgel system was found to be in-sensitive to pH changes over the wide pH range (from pH = 2 to pH = 10) due to absence of ionizable groups in P(VCL-AAEM) microgels. The pH responsiveness was found to be dependent on content of ionizable moiety (VIM) present in P(VCL-AAEM-VIM) microgels because of increase of electrostatic repulsions caused by increase in ionizable groups. Moreover, VPTT of the P(VCL-AAEM-VIM) microgels at pH = 4 was found to be much higher than that at pH = 6 in aqueous medium. Agrawal *et al.*^24^ synthesized another pH responsive microgel system by polymerization of VCL and AAEM with acrylic acid (AA) to obtain P(VCL-AAEM-AA) microgels. In contrast to VIM, polymerization of VCL and AAEM with AA monomer may give a pH responsive microgel which swell upon increase of pH of the medium. P(VCL-AAEM-AA) microgels exits in swollen state at pH ≥ 4.2 due to electrostatic repulsion caused by ionization of –COOH groups of acrylic acid moieties of the microgels to –COO^−^ groups and in shrunken state at pH ≤ 4.2 due to protonation of COO^−^ to form –COOH groups that have no electrostatic repulsion. Au nanoparticles were loaded into P(VCL-AAEM-AA) microgels to obtain pH responsive hybrid microgels but detailed pH responsive behavior of the microgels and hybrid microgels was not investigated by this group because aim of their study was to investigate the catalytic activity of Au-P(VCL-AAEM-AA) hybrid microgels. The investigation of pH responsive behavior and pH dependent optical properties of Au-P(VCL-AAEM-AA) hybrid microgels may be an interesting future study. Copolymerization of VCL with 4-vinyl pyridine (4VP) in the presence of cross-linker may produce another VCL based pH responsive microgel system. P(VCL-*co*-4VP) microgel systems have also been found to be responsive towards pH as decrease in pH resulted in the protonation of pyridine rings in 4VP.^27^ They fabricated Au nanoparticles inside the P(VCL-*co*-4VP) microgels and found that presence of Au nanoparticles does not affect responsive behavior of the microgels. Moreover optical properties of Au nanoparticles may be tuned by changing the pH and temperature of the medium.

### 5.2 Properties of VCL based hybrid microgels due to inorganic component

#### 5.2.1 Optical properties of VCL based hybrid microgels

Various inorganic nanoparticles like gold^70^ and silver^71^ NPs loaded into microgels are known for their fascinating optical properties. Optical properties of inorganic nanoparticles loaded into smart polymer microgels can be easily tuned by changing the environmental stimuli. Actually optical properties of inorganic nanoparticles depend upon their size, shape, refractive index of the medium and inter-particles distance. Change in external stimulus causes swelling/deswelling in polymer microgel particles which results in change of refractive index around nanoparticles and inter-nanoparticles distance.^72^ As a result, optical properties of nanoparticles can be manipulated which is highly useful for various sensing based applications.^73,74^ Various inorganic nanoparticles having tunable optical properties have been loaded into VCL based microgels.^24,27,45,63,64^ Fabrication, characterization and applications of optical nanomaterial based hybrid microgels made of VCL have been properly discussed but tuning of optical properties by external stimuli has not been explored in detail. For example, Agrawal *et al.*^24^ have fabricated and characterized Au nanoparticles *in situ* generated in P(VCL-AAEM-AA) microgels for catalytic applications but study of optical properties has not been reported. Study of surface plasmon resonance wavelength of gold nanoparticles loaded into P(VCL-AAEM-AA) microgels as a function of medium temperature will be an interesting aspect of this hybrid system and may be explored in future investigations. Hantzschel *et al.*^48^ reported silver nanoparticles having optical properties in VCL based microgels for their antibacterial applications but did not study the optical properties of silver nanoparticles in detail. Shape, size and size distribution of silver nanoparticles loaded into VCL based microgels may be investigated using surface plasmon resonance property. Moreover growth of silver nanoparticles during their fabrication within microgels and their stability over the time may be studied by UV-visible spectroscopy based on their optical properties. Moreover, study of surface plasmon resonance wavelength as function of temperature will be interesting which has not been reported this particular hybrid system. Pich *et al.*^64^ studied measured wavelength of maximum absorption as a function of size of ZnS nanoparticles loaded into P(VCL-AAEM) microgels and noted that value of wavelength of maximum absorption is red shifted with increase of size of nanoparticles but wavelength of maximum absorption of ZnS loaded in temperature responsive VCL based microgels as a function of temperature of the medium was not investigated. Study of optical properties of ZnS nanoparticles as a function of temperature will be a step towards designing of a new optical temperature sensor based on ZnS-P(VCL-AAEM) hybrid microgels. Peng *et al.*^25^ synthesized a series of P(VCL-MAA)-CuS hybrid microgels with different COO^−^/Cu^2+^ ratios and studied surface plasmon resonance property of CuS nanocrystals as a function of content of CuS precursors and pH of medium. Among them, P(VCL-MAA)-CuS hybrid microgel exhibited distinct absorption band at 965 nm due to high-density free carriers in covellite nanocrystals of CuS. The value of surface plasmon resonance wavelength was found tunable with precursor content and pH of the medium. Siirila *et al.*^58^ reported surface decoration of P(VCL) based nanogels with Au nanoparticles and studied optical properties of Au nanoparticles. Surface plasmon resonance (SPR) of the Au nanoparticles dispersed in ethanol and in 10 mM aqueous 4-(2-hydroxyethyl)-1-piperazineethanesulfonic acid) (HEPES) was determined using UV-vis spectroscopy. Surface plasmon resonance peak found to be at 522 nm in ethanol and at 528 nm in 10 mM HEPES. Obtained SPR values confirmed truncated octahedral shape of the Au nanoparticles.

#### 5.2.2 Magnetic properties of VCL based hybrid microgels

Magnetic properties of hybrid microgels are of high importance with respect to their biomedical^75^ and catalytic^76^ applications. Therefore fabrication of magnetic nanoparticles in VCL based microgels has been reported in literature.^33,77^ Pich *et al.*^33^ fabricated Fe_3_O_4_ nanoparticles in P(VCL-AAEM) microgels and investigated their magnetic properties. It was found that resulting Fe_3_O_4_-P(VCL-AAEM) hybrid microgels have magnetic properties along with temperature sensitivity. Magnetization was plotted against applied magnetic field for determination of magnetization saturation value which was found dependent of Fe_3_O_4_ content loaded into the microgels. The value of saturation magnetization of Fe_3_O_4_-P(VCL-AAEM) hybrid microgels was increased with increase of Fe_3_O_4_ content in microgel system. The fabrication of catalytically active nanoparticles in Fe_3_O_4_-P(VCL-AAEM) hybrid microgels will give a thermally tunable and magnetically separable catalytic system. Medeiros *et al.*^77^ synthesized poly(*N*-vinylcaprolactam-*co*-itaconic acid) – [P(VCL-*co*-IA)] microgels using Fe_3_O_4_ nanoparticles as seed magnetic nanoparticles for development of drug delivery to be monitored by magnetic resonance imaging. Resulting hybrid material was found to have pH-sensitivity, temperature sensitivity and magnetic properties.

## 6.0 Catalytic applications of VCL based hybrid microgels

Inorganic nanoparticles loaded into VCL based microgels have a potential to be used as catalysts for various organic reactions. Catalytic applications of VCL based hybrid microgels towards various reactions has been discussed here.

### 6.1 Catalytic reduction of nitroarenes

Catalytic reduction of various nitroarenes in the presence of hybrid microgels has been widely reported in literature but reduction of 4-nitrophenol (4-NP) to 4-aminophenol (4-AP) has become a bench mark reaction to exam the catalytic ability of hybrid microgels. Attention towards reduction of 4-NP is stimulated by various reasons. 4-NP is released from various industries and has become a source of water pollution while reduction product, 4-AP is less toxic and useful reaction intermediates of various industries.^78,79^ The progress of the reaction can be easily examined spectrophotometrically. Pich *et al.*^56^ investigated catalytic efficacy of Au-P(VCL-*co*-AAEM) hybrid microgels towards reduction of 4-nitrophenol by sodium borohydride under various reaction conditions in aqueous medium. The progress of the reaction was observed by UV-Vis spectrophotometry. UV visible spectra of reaction mixture containing 4-NP, NaBH_4_ and Au-P(VCL-AAEM) hybrid microgels as a function of time was scanned in wavelength range of 190–820 nm. Decrease in absorbance of peak at 400 nm along with increase in absorbance of peak at 300 nm with time was noted due to consumption of 4-NP and formation of 4-AP. Pseudo first order kinetic model was applied for evaluation of apparent rate constant (*k*_app_) for reduction of 4-NP under various reaction conditions. The concentration of NaBH_4_ was kept much higher than that of 4-NP under all experiments to fulfill the requirement of pseudo first order reaction. The value of *k*_app_ was found to be increased with increase of concentration of hybrid microgels and Au content in hybrid microgels which indicated that reaction occurs on the surface of Au nanoparticles loaded into microgels. Value of rate constant for Au-P(VCL-AAEM) catalyzed 4-NP reduction was found to be higher than that in the presence of same amount of naked Au nanoparticles under similar conditions which was attributed to aggregation of naked Au nanoparticles during catalysis. The increase in temperature resulted in decrease in reduction rate due to diffusional barrier caused by shrinkage of P(VCL-AAEM) network. The value of rate constant of Au-P(VCL-AAEM) catalyzed reaction was found to be increased with further increase of temperature due to shift of Au nanoparticles from core of microgel particles to microgel–water interface. The values of activation energy of Au-P(VCL-AAEM) catalyzed reduction of 4-NP and naked Au nanoparticles catalyzed 4-NP reduction were found to be 36 kJ mol^−1^ and 71 kJ mol^−1^ respectively. Low activation energy in case of hybrid microgel catalyzed reaction was an indication of role of microgel in facilitation of conversion of 4-NP to 4-AP on the surface of Au nanoparticles. Rate of catalytic reduction of 4-NP can be increased by using Au nanoparticles stabilized in specially modified VCL based microgels. Jia *et al.*^46^ have reported enhanced catalytic activity of Au-P(VCL-α-CD) hybrid microgels in comparison to un-modified Au-P(VCL-AAEM) hybrid microgels. They reported that α-CD has ability to capture 4-NP from aqueous medium because of host–guest interactions. The α-CD forms complexes with 4-NP and increases the surface concentration of 4-NP. In other words α-CD facilitates the adsorption of 4-NP on the surface of Au nanoparticles. Therefore value of rate constant for reduction of 4-NP in the presence of Au-P(VCL-α-CD) hybrid microgels was found to be higher than that in the presence of Au-P(VCL-AAEM) hybrid microgels. The product 4-AP cannot form a complex with α-CD. Hence 4-AP molecules desorb from Au surface to vacate adsorption sites for 4-NP for further reaction. Interestingly the catalytic system was found to be selective for reduction of 4-NP in comparison to 2,6-dimethyl-4-nitrophenol under similar conditions because α-CD cannot accommodate bulky 2,6-dimethyl-4-nitrophenol in its cavity. Similarly hybrid microgels having hydrophilic moieties in their network will be favorable for rapid diffusion of hydrophilic monomers from bulk region to catalyst surface which results in increase of rate of catalytic reaction. Agrawal *et al.*^24^ have tested the catalytic activity of Au-P(VCL-AAEM-AA) hybrid microgels having hydrophilic moiety (AA) towards reduction of 4-NP in aqueous medium. The presence of AA in VCL based hybrid microgels enhances the rate of catalytic reduction of 4-NP in two ways. AA is hydrophilic and has affinity towards 4-nitrophenolate ions. Catalytic reduction of 4-NP by NaBH_4_ is carried out in basic pH where carboxylic acid groups of AA exist in the form of carboxylate ions due to which the microgel system exist in swollen state. Therefore reactant molecules may access the surface of Au nanoparticles without any substantial diffusional obstacle. Catalytic reduction of 4-NP using VCL based hybrid microgels as catalyst has been also reported by some other groups using the same methodology as described above and may be studied from recent literature.^80,81^

### 6. 2 Catalytic degradation of organic dye

Organic dyes are highly toxic substances that are released from various industries and have become a source of water pollution. Removal of dyes from aqueous medium by different smart polymer microgels and hybrid microgels is a subject of intensive research and a plenty of work has been reported by us^81–83^ and others.^84,85^ But literature on VCL based hybrid microgels catalyzed reduction/degradation of organic dyes is limited. Siddiq *et al.*^80^ have made an attempt to catalyze reduction of methyl orange (MO) and methylene blue (MB) with sodium borohydride using Silver-poly(vinylcaprolactam-*co*-itaconic acid) [Ag-P(VCL-IA)] hybrid microgels as a catalyst in aqueous medium. Progress of reduction of both dyes was observed spectrophotometrically. Pseudo first order kinetic modeling was applied for evaluation of rate constant of catalytic reduction of dyes in the presence of Ag-P(VCL-IA) hybrid microgels. The value of rate constant for reduction of dye was found to be dependent of catalyst dose and temperature. Mechanism of reduction of dyes was proposed on the basis of experimental observation. Dye molecules are adsorbed on the surface of nanoparticles and interact with BH_4_^−^ to get reduce. The products desorb from catalyst surface and diffuse out towards bulk region. Identification of reaction intermediates and degradation products formed during catalytic reduction of dyes needs further investigation. Recovery of the catalyst from reaction mixture, investigation of changes occurred in morphology and structures of hybrid microgels after catalysis and re-use was not explored which is essential for economical catalytic process.

### 6.3 Suzuki coupling reactions

**Scheme 1:**
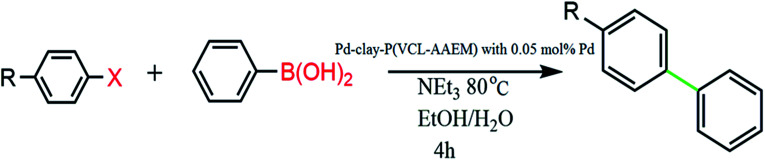
Pd-clay-P(VCL-AAEM) catalyzed Suzuki coupling reaction in water–ethanol medium at 80 °C.

Pd nanoparticles stabilized polymer microgels are widely used as catalysts for Suzuki coupling reactions but only few reports are available on stabilization of Pd nanoparticles in VCL based polymer microgels for such kind of organic transformation.^57,86,87^ Contin *et al.*^57^ reported catalytic ability of Pd-clay-P(VCL-AAEM) hybrid microgels towards Suzuki coupling reactions using organic base, trimethylamine (NEt_3_) in water–ethanol (EtOH/H_2_O) medium at 80 °C according to Scheme 1.

**Scheme 2:**
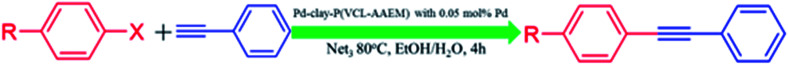
Pd-clay-P(VCL-AAEM) catalyzed Sonogashira coupling reaction in water–ethanol medium at 80 °C.

During optimization of above mentioned conditions, flocculation of hybrid microgels was observed in case of Suzuki coupling reaction in the presence of inorganic base (NaOH and K_2_CO_3_). Pd-clay-P(VCL-AAEM) hybrid microgels were found to be highly efficient catalyst for Suzuki coupling of a wide variety of aryl halides with different X and R in water–ethanol mixture under mild conditions. The yield of the product was found to be good to excellent in most of the cases. They also tried Sonogashira coupling reaction shown in Scheme 2 under similar reaction conditions but Pd-clay-P(VCL-AAEM) hybrid microgels did not show any activity towards copper free Sonogashira reactions.

Catalytic activity of Pd-clay-P(VCL-AAEM) hybrid microgels towards Suzuki coupling reaction was found to dependent on size of Pd nanoparticles and content of clay in Pd-clay-P(VCL-AAEM) hybrid microgels. Role of clay in enhancement of catalytic activity needs further studies.

**Scheme 3:**
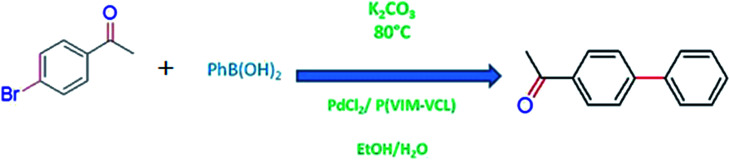
Palladium nanoparticles loaded into VCL based microgels catalyzed coupling reaction in water–ethanol medium at 80 °C.

Selivanova *et al.*^86^ reported Pd-P(VIM-VCL) hybrid microgel as an effective and recyclable catalyst for the Suzuki coupling reactions under optimal conditions of 1 : 1 EtOH/H_2_O with K_2_CO_3_ at 80 °C according to Scheme 3.

Above mentioned optimum conditions has been observed in highest stability and activity of catalyst. Suzuki reaction was also performed for a range of aryl bromides containing electron donor and acceptor substituents.

**Scheme 4:**
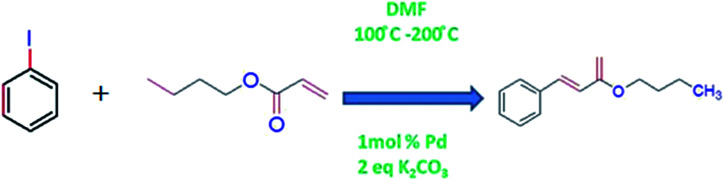
Palladium nanoparticles loaded into VCL based microgels catalyzed coupling reaction in water–ethanol medium at 80 °C.

Beletskaya *et al.*^87^ reported efficient catalytic activity of palladium supported on poly(*N*-vinylimidazole) [P(VIM)] and poly(*N*-vinylimidazole-*co-N*-vinylcaprolactam) [P(VIM-VCL)] towards Mizoroki–Heck model reaction using phenyl iodide and *n*-butyl acrylate as reactants in (DMF) in the presence of K_2_CO_3_ base and 1 mol% Pd according to Scheme 4.

The reaction rate was found to be faster in the presence of Pd-P(VIM-VCL) hybrid microgels. They also investigated catalytic efficiency of the hybrid system for a number of aryl iodides and aryl bromides being used as substrates. In each case, products were formed in high yield and good stereo selectivity.

## 7.0 Biomedical application of VCL based hybrid microgels

**Fig. 5:**
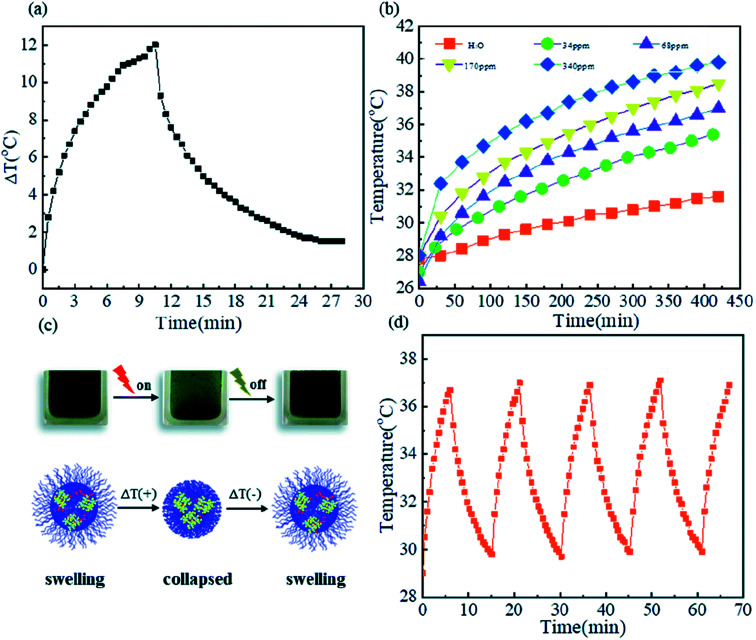
(a) Change in temperature of poly(*N*-vinylcaplrolactam-*co*-methacrylic acid)-CuS hybrid microgels with 68 ppm Cu in aqueous medium as a function of time upon illumination with 980 nm laser (0.67 W cm^−2^) for 10 minutes, (b) temperature of poly(*N*-vinylcaplrolactam-*co*-methacrylic acid) microgels loaded without and with different CuS contents as function of time of illumination with 980 nm laser (0.67 W cm^−2^), (c) schematic illustration of photo-thermal effect caused by swelling–deswelling of poly(*N*-vinylcaplrolactam-*co*-methacrylic acid)-CuS hybrid microgels upon irradiation, (d) temperature–time curve of poly(*N*-vinylcaplrolactam-*co*-methacrylic acid)-CuS hybrid microgels with 68 ppm CuS during five ON/OFF cycles of same laser. These images have been reproduced from ref. 25. Copyright 2020 Royal Society of Chemistry.

Microgels have been investigated extensively to be used in biomedical field due to their tunable size and temperature/pH responsive properties. Among temperature sensitive microgels, NIPAAm based microgel systems are most extensively investigated for potential biomedical applications but their practical applicability becomes limited due to neurotoxicity of NIPAAm. In contrast to NIPAAm, VCL based microgels have been developed which have gained more attraction as biological compatible polymeric systems because VCL is less toxic and upon hydrolysis, amide group of P(VCL) does not produce small amide compounds that are toxic. But unlike extensive literature on NIPAAm based microgels,^74,88^ a limited reports are available on potential biomedical applications of VCL based responsive microgels.^89–91^ Biomedical applications of VCL based microgels have been already reviewed in literature.^90^ Therefore our discussion is limited to biomedical potential of VCL based microgels loaded with inorganic nanoparticles. Medeiros *et al.*^77^ have reported poly(*N*-vinylcaprolactam-itaconic acid) microgels loaded with Fe_3_O_4_ nanoparticles as a pH, temperature and magnetic field responsive drug carrier system. The system was found biocompatible but their practical utility was not studied in detail. Metal sulfide nanoparticles loaded VCL based microgels may also be used as drug delivery system. For example, Peng *et al.*^25^ have recently reported CuS nanocrystals loaded into poly(*N*-vinylcaprolactam-*co*-methacrylic acid) microgel for photo-thermal cancer therapy. Plasmonic CuS nanocrystals loaded into microgels have ability to absorb near infrared radiations and convert light energy into heat which is used to cause deswelling in microgels. As a result of deswelling of hybrid microgels, drug is released to the target site. An important feature of this system is the control over un-expected harm. When temperature is increased then hybrid microgel particles get de-swelled. As a result, absorption of light by CuS becomes difficult due to barrier caused by polymer network around CuS crystals which causes drop in temperature again. Change in temperature of poly(*N*-vinylcaplrolactam-*co*-methacrylic acid)-CuS hybrid microgels with 68 ppm Cu in aqueous medium as a function of time upon illumination with 980 nm laser (0.67 W cm^−2^) for 10 minutes is shown in Fig. 5(a). The photo-thermal effect measured in the form of temperature profile (*T*–*t* curve) also depends upon content of CuS in microgel as shown in Fig. 5(b). P(VCL-MAA)@CuS hybrid microgel system is safe from un-expected tissue harm as illustrated in Fig. 5(c). P(VCL-MAA)@CuS hybrid microgel system was found to be stable over various on/off cycles as shown in Fig. 5(d).

## 8.0 Conclusion and future perspective

VCL based hybrid microgels have fascinating optical, magnetic and catalytic properties depending upon their inorganic component along with responsive nature of organic component and have become an emerging field in research. This review has spotlight on brief survey regarding synthesis, characterization, properties and applications of VCL based hybrid microgels. Surfactant free emulsion polymerization was found to be the most effective among polymerization techniques for synthesizing VCL based microgels. Various inorganic nanoparticles including Au, Ag, FePt, ZnO, ZnS, LaF_3_:Eu and Fe_3_O_4_ nanoparticles have been incorporated inside the VCL based microgel systems to add some additional properties into the systems for different applications. Characterization techniques like SEM, TEM, DLS, NMR, FTIR and UV-Vis *etc.* have been summarized to study the properties and behavior of hybrid microgels. VCL based hybrid microgels have a potential to be used as catalysts for various organic reactions. We believe that VCL based hybrid system will gain much attention in future due to its fascinating characteristics. Au and Ag NPs are most frequently incorporated NPs inside the VCL based microgels. Both Au and Ag nanoparticles are plasmonic in nature and have interesting optical properties. Tuning of optical properties of plasmonic nanoparticles loaded into VCL based microgels by varying the environmental conditions to develop new sensors. In addition to Au and Ag nanoparticles, other metal nanoparticles like Pt, Pd, Rh, Ni, Cu and Co nanoparticles also need to be employed and stabilized inside the network. VCL based microgels loaded with Fe_3_O_4_ nanoparticles have been synthesized and characterized.^33^ They may be used as micro-reactors to fabricate metal nanoparticles to design magnetically separable catalytic system for different organic transformations. Responsive properties of VCL based microgels are declined by the incorporation of inorganic nanoparticles. The ratio of inorganic and microgel contents should be optimized to obtain hybrid microgels with properties of both components in a single system. To the best of our knowledge, only catalytic reduction of 4-NP and 2, 6-dimethyl-4-nitrophenol from a wide variety of nitroarenes using VCL based hybrid microgels has been reported in literature. The catalytic activity of VCL based hybrid system must be extended towards reduction of various nitroarenes. Moreover, resultant aminoarenes must be separated, purified and characterized which is highly important for synthetic organic chemistry point of view. VCL based hybrid microgels have been reported for catalytic reduction of only two dyes (MO and MB). Catalytic degradation of other organic dyes like rhodamine B, Eosin and Congo red *etc.* may be carried out in the presence of VCL based hybrid microgels. VCL based hybrid microgels may be used for different organic transformations including hydrogenation, cyclization, reduction, oxidation, amination and many more. The recyclability and reusability of VCL based hybrid microgels along with investigation of changes occurring in catalytic systems may be a subject of future publications. Beside catalytic applications, VCL based microgels have also been reported to be used as a potential biocompatible polymer for drug delivery. This drug system needs to be modified into photo responsive system for future publications. Kumacheva *et al.*^74^ have loaded Au rods in P(Nipam-AA) for photo responsive drug delivery system but Nipam is toxic. Au nano rods having longitudinal surface plasmon resonance in water window region may be loaded in VCL based microgels to design nontoxic VCL based photo responsive drug delivery system.

## Abbreviations

AA: Acrylic acid
AAEM: Acetoacetoxyethyl methacrylate
AAS: Atom absorption spectroscopy
ACMA: 2,2′-Azobis [*N*-(2-carboxyethyl)-2-methylpropionamidine]
AEP: 2-Aminoethyl dihydrogenphosphate
AgNO_3_: Silver nitrate
Ag NPs: Silver nanoparticles
Ag-P(VCL-IA): Silver-poly(vinylcaprolactam-*co*-itaconic acid)
AFM: Atomic force microscopy
AMPA: 2,2′-Azobis(2-methylpropioamidine) dihydrochloride
4-AP: 4-Aminophenol
Au NPs: Gold nanoparticles
Au-P(VCL-AAEM): Gold-poly[vinylcaprolactam-*co*-(acetoacetoxyethyl methacrylate)]
Au-P(VCL-α-CD): Gold-poly[vinylcaprolactam-*co*-α-cyclodextrin]
AZT: 11-Azidoundecanethiol
α-CD: α-Cyclodextrin
BAC: *N*,*N*-Bis(acryloyl) cystamine
CTAB: Cetyl trimethyl ammonium bromide
DLS: Dynamic light scattering
DMF: *N*,*N*-Dimethylformamide
DOX: Doxorubicin
EDX: Energy dispersive X-ray
FTIR: Fourier Transform Infrared Microscopy
GMA: Glycidyl methacrylate
GSH: Glutathione
HEPES: 4-(2-hydroxyethyl)-1-piperazineethanesulfonic acid)
IA: Itaconic acid
KPS: Potassium persulfate
MA: Methacrylic acid
MBA: *N*,*N*′-Methylenebisacrylamide
MB: Methylene blue
MO: Methyl orange
MUA: 11-Mercaptoundecanoicacid
NEt_3_: Trimethylamine
4-NP: 4-Nitrophenol
NPs: Nanoparticles
NIPAAm: *N*-Isopropylacrylamide
PEGDA: Poly(ethylene glycol) diacrylate
PEGMA: Polyethylene glycol methyl ether methacrylate
P(VCL-AAEM): Poly[vinylcaprolactam-*co*-(acetoacetoxyethyl methacrylate)]
P(VCL-AAEM-AA): Poly(*N*-vinylcaprolactam-*co*-acetoacetoxyethyl methacrylate-*co*-acrylic acid)
P(VCL-AAEM-VIM): Poly(vinylcaprolactam-*co*-acetoacetoxyethyl methacrylate-*co*-vinylimidazole)
P(VCL-*co*-AGA): Poly(*N*-vinylcaprolactam-co acrylamidoglycolic acid)
P(VCL-*co*-IA): Poly(*N*-vinylcaprolactam-*co*-itaconic acid)
P(VCL-*co*-MA): Poly(*N*-vinyl caprolactam-*co*-methacrylic acid)
P(VCL-GMA): Poly(*N*-vinylcaprolactam-*co*-glycidyl methacrylate)
P(VCL-*co*-UA): Poly(*N*-vinylcaprolactam-*co*-undecenoic acid)
P(VCL-*co*-4VP): Poly(*N*-vinylcaprolactam-*co*-4-vinyl pyridine)
P(VIM-VCL): Poly(*N*-vinylimidazole-*co-N*-vinylcaprolactam)
Py: Pyrrole
*R*_h_
: Hydrodynamic radius
SDS: Sodiumdodecyl sulphate
SEM: Scanning electron microscopy
SPR: Surface plasmon resonance
TAA: Thioacetamide
TEA: 2-Aminoethanthiol
TEM: Transmission electron microscopy
TGA: Thermogravimetric analysis
THF: Tetrahydrofurane
UV/Vis: UV-visible spectroscopy
VCL: *N*-Vinyl caprolactam
VPTT: Volume phase transition temperature
VIM: Vinylimidazole
VP: Vinyl pyridine
XPS: X-ray photoelectron spectroscopy
XRD: X-ray diffraction technique
ZnAc: Zinc acetate

## Conflicts of interest

Authors declare no conflict of interest.

## Supplementary Material
